# A Mobile and Low-Cost System for Environmental Monitoring: A Case Study

**DOI:** 10.3390/s16050710

**Published:** 2016-05-17

**Authors:** Alejandro Velasco, Renato Ferrero, Filippo Gandino, Bartolomeo Montrucchio, Maurizio Rebaudengo

**Affiliations:** DAUIN, Politecnico di Torino, Corso Duca degli Abruzzi, 24, 10129 Torino, Italy; alejandro.velasco@polito.it (A.V.); filippo.gandino@polito.it (F.G.); bartolomeo.montrucchio@polito.it (B.M.); maurizio.rebaudengo@polito.it (M.R.)

**Keywords:** air monitoring, air pollution, wireless sensor networks, mobile sensors

## Abstract

Northern Italy has one of the highest air pollution levels in the European Union. This paper describes a mobile wireless sensor network system intended to complement the already existing official air quality monitoring systems of the metropolitan town of Torino. The system is characterized by a high portability and low cost, in both acquisition and maintenance. The high portability of the system aims to improve the spatial distribution and resolution of the measurements from the official static monitoring stations. Commercial PM${}_{10}$ and O${}_{3}$ sensors were incorporated into the system and were subsequently tested in a controlled environment and in the field. The test in the field, performed in collaboration with the local environmental agency, revealed that the sensors can provide accurate data if properly calibrated and maintained. Further tests were carried out by mounting the system on bicycles in order to increase their mobility.

## 1. Introduction

Air-quality has a huge impact on the quality of life, and long-term exposure to polluted air can result in permanent health issues [1,2]. In agriculture, air pollution has a great impact on crop yields by causing visible injury symptoms to foliage, thus affecting the economic development of a region [3]. For these reasons, air quality monitoring is required by national air quality regulations, such as the European directives 2008/50/EC and 2004/107/CE. The equipment necessary to meet the standards established by these regulations in order to monitor air quality has a high cost of procurement and maintenance. For example, the purchase and installation of a single gas-analyzer in already existing infrastructures can cost between £10,000 and £15,000 [4].

In recent years, many projects have been developed in order to provide less expensive solutions to air quality monitoring [5,6]. The implementation of large quantities of low-cost sensors in a wireless network can increase the coverage area and spatial distribution of the monitoring systems, especially if mounted on mobile platforms [7]. These low-cost devices are not meant to replace official air control systems, but to integrate their readings [8]. These devices can be autonomous and equipped with power-generating capabilities [9], in addition to adaptable communication protocols [10,11].

This paper presents a proposal to implement such systems in one of the most critical areas in the whole European Union according to the European Environment Agency [12]: the city of Torino in the northwest of Italy. The proposed low-cost mobile system can be mounted on a public bike sharing system and is based on the platform presented in [13]. The presented solution would complement official monitoring systems by monitoring the levels of certain pollutants, such as O${}_{3}$ and PM${}_{10}$, with a less accurate and less expensive equipment, but increasing the spatial distribution of readings aiming to achieve a street-level resolution. Additionally, the study identifies possible scenarios in which these types of sensors can be used to effectively enhance environmental monitoring by extending the area under study. The platform must therefore be able to accept data from both fixed stations and mobile nodes and must have the ability to associate each measured value with the correct georeference, by means of a GPS unit, and to auto-calibrate the sensors.

## 2. Related Works

Air pollution monitoring is a common application field for wireless sensor network (WSN) systems. For example, WSNs are used to assess the impact on the environment of locations, such as mines [14] and airports [15], to detect critical events, such as fires [16], and to estimate their effects, such as the emission release of noxious gases [17]. The improvement of systems for wide-area monitoring has became a trend over the last few years, thanks to new technologies and miniaturization of components with significant processing power. This trend is leading towards low-cost, low-energy and mobile nodes [18]. All of these characteristics are exploited in the current study.

Low-cost WSNs can be used as an independent platform or as a complementary one to existing systems. The first scenario is feasible with a proper configuration of low-cost sensors, because in this way, sensors commonly used for measuring at the parts-per-million (ppm) level can provide reliable results in the parts-per-billion (ppb) scale [19]. Evidence of the second scenario can be seen in government-funded projects over the last decade. For example, the European Commission funded a project called “Open architecture for Smart and Interoperable networks in Risk management based on In-situ Sensors” (OSIRIS) [20]. This project developed smart wireless networks that can be used to monitor air pollution, water pollution and emissions from forest wildfires and industrial fires. The development of these smart wireless sensor networks was aimed to complement measurements of ground stations and aircraft.

Low-energy WSNs have been proven reliable for environment monitoring, for example in detecting gas leaks in buildings [21]. Similarly, the power-saving capability of a system for monitoring PM${}_{10}$ and carbon monoxide is able to guarantee a prolonged operating time, by means of solar panels and batteries [9]. Power savings can also be achieved by implementing algorithms to reduce the amount of transferred data [22].

Mobile sensors are commonly exploited for monitoring metropolitan areas. The nodes can be put on board vehicles, such as private cars [23] or public transport systems [7], by using unmanned aerial vehicle (UAV) [24], or they can be a personal sensing device [7]. In addition, relying on bicycles to build a monitoring network has proven possible [25,26]. Both public (*i.e.*, available for sharing) and private (*i.e.*, *ad hoc* designed) bicycles can be used. Public bicycles can be equipped with a node consisting of a processor, a sensor to detect exhaust gas, a GPS receiver to track the position, a microSD card to store the collected data and a Bluetooth module to upload the data when the bicycle is parked at the docking station [25]. Private bicycles can host more sensors, such as ultra-fine particles, PM${}_{10}$, black carbon and CO, and they can provide automated data transmission, data pre-processing and data visualization [26].

## 3. Complementary Mobile Sensing System

The aim of this project is to develop a low-cost system capable of acquiring multiple data on the move, at acceptable costs. This would complement an existing monitoring system by increasing the spatial distribution of the collected data. Various scenarios in which these types of sensors may be useful and effective have been tested.

The study takes place in the city of Torino, which is located in Northern Italy, one of the areas with the worst air quality in the European Union [12]. Here, the official entity in charge of air quality monitoring is ARPA Piemonte (“Agenzia Regionale per la Protezione dell’Ambiente del Piemonte”). It currently has a total of 17 fixed monitoring stations in the province of Torino, among which five are located in the city of Torino. These five stations provide a coverage area of 130 km${}^{2}$: this is the area with the highest population density in the region of Piemonte, reaching 900,000 inhabitants. As previously mentioned, the situation for the northern regions of Italy has become critical, and extraordinary measures have been taken in order to counteract air pollution, such as the enforcement of a complete ban of road traffic during the day and providing free public transportation on critical days to encourage people to leave their motor vehicle at home. A low cost monitoring system on the public bike sharing scheme has been proposed in order to provide the authorities and the population with more data, especially with higher spatial resolution.

### 3.1. Proposed Implementation

**Figure 1:**
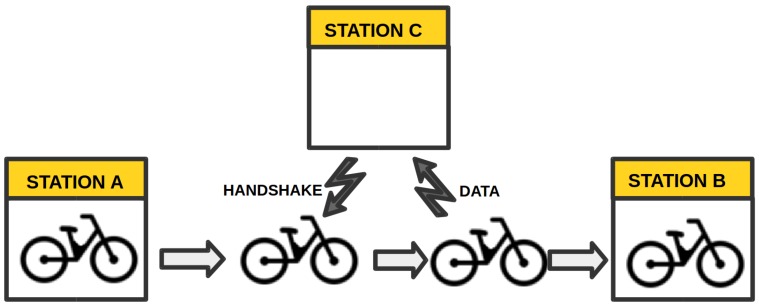
Proposed system’s operation diagram.

The system proposed in this study could take advantage of the trips made by cyclists and of the coverage of the stations of the public bike sharing system. For example, the bike sharing system of the city of Torino, which is called “TO” bike, has 116 docking stations capable of docking a dozen bikes each. Some of the stations and bikes could be equipped with a low cost monitoring system capable of RF communications and GPS, in the case of the stations also with an RF hub for a selected communication protocol. As the bikes equipped with sensors move through the city, they could collect data and then relay these data to the docking stations equipped with wireless hubs; then, these stations could subsequently communicate the data to the main server via an Internet connection. The hubs at the stations would be constantly transmitting a handshake request, so that if any bike equipped with sensors passes close to these stations, they request the data collected. The data collected by the bikes could be paired with the georeference provided by the GPS, so that pollutants can be associated with a particular street. Figure 1 provides a general idea of the possible operation of the system: at first, the bike mounted with sensors is at Station A; then, it is taken and transits towards Station B. While moving, the bike gets near Station C and responds to the handshake transmitted by the station: after the RF link has been established, the sensors could then send the data collected to the station.

Since low-cost sensors need to be constantly calibrated [27], an additional hub could be installed in one of the ARPA data collection sites. It would allow the transmission of the current registered values of the pollutants being monitored by the proposed system. Then, by taking these values and the current values being measured by the mobile system, an *in situ* calibration could be performed, thus saving time for calibration. A similar approach could be adopted by mounting the sensors on bikes: each time two bikes get close enough to get a handshake with a comparison of the last calibration time, then the bike with the most recent calibration could transmit the value read, and the receiving bike would calibrate its sensors. Finally, when data are transmitted at the docking stations, and stored on the server, they should be post-processed by taking the mean values hourly in order to counteract any possible erroneous data provided by the measurement drifts of the sensors.

### 3.2. System Hardware

Different candidates for the monitoring system have been evaluated in [13]. The capability of the different systems to be used for monitoring different pollutants was considered as a decisive feature. An important feature to be considered is the cost of the system: it should be notably lower than the cost of existing platforms, normally characterized by high purchase costs [4] and significant maintenance expenses. Basic data acquisition, by connecting the system to existing platforms through open source software, is considered as a further potential advantage.

**Figure 2:**
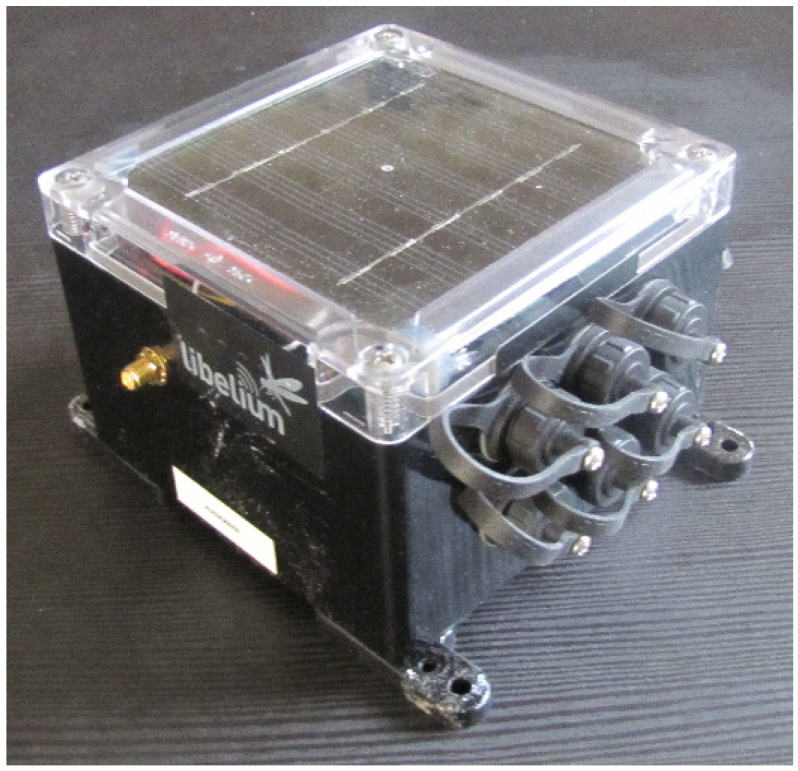
Waspmote Plug & Sense platform.

Table 1 shows the different platforms taken into consideration in [13] and some of the parameters considered for the final selection. The “Waspmote Plug & Sense” system, displayed in Figure 2 and hereinafter called Waspmote, can ensure the low cost, portability and reliability features required of a mobile system. In fact, Waspmote sensors are successfully exploited in existing air monitoring systems [28]. The considered pollutants are O${}_{3}$ and fine dust (PM${}_{10}$). Data were obtained in various locations: indoors and outdoors, urban and rural locations, with mobile and static stations. Indoor and control tests were carried out in the beginning of 2015, while outdoor tests were performed when the parameters present their peak concentration, according to historical data: O${}_{3}$ was measured during the summer of 2015 and PM${}_{10}$ during the autumn of 2015 and summer of 2016.

### 3.3. Sensors’ Specifications

The PM${}_{10}$ sensor implemented in this study corresponds to the GPY21010AU0F sensor manufactured by Sharp Co. [29]. The sensor evaluates the concentration of PM${}_{10}$ in the atmosphere by measuring the dust induced scattering of light from an LED.

- Operational range: 0–0.5 mg/m${}^{3}$
- Sensitivity: 0.5 V (0.1 mg/m${}^{3}$).

The ozone sensor exploited in this study is MiCS-2610, manufactured by e2v Technologies Ltd. [30]. It evaluates the concentration of ozone in the atmosphere by measuring the voltage in the arrangement of two internal resistors: the first acts as a reference and the other as a variable resistor sensitive to ozone.

- Operational range: 10–1000 ppb
- Sensitivity: 2–4 ohm
- Response time: 30 s.

The two sensors have been mounted on the Waspmote platform. In addition, the Waspmote owns an internal GPS, based on the JN3 chip from Telit Communications PLC [31]. Its characteristics are:

- Acquisition sensitivity: −147 dBm
- Navigation sensitivity: −160 dBm
- Tracking sensitivity: −163 dBm
- Hot start time: <1 s
- Cold start Time: <35 s
- Positional accuracy error: <2.5 m
- Speed accuracy: <0.01 m/s.

## 4. Wireless Networks

As referred to in Section 3, the Waspmote can use different wireless communication protocols options, such as 802.15.4, ZigBee, Wi-Fi (802.11b/g), 3G/GPRS and BLE (Bluetooth Low Energy). Several tests were conducted to evaluate the system, in particular considering ZigBee, Wi-Fi and BLE.

**Figure 3:**
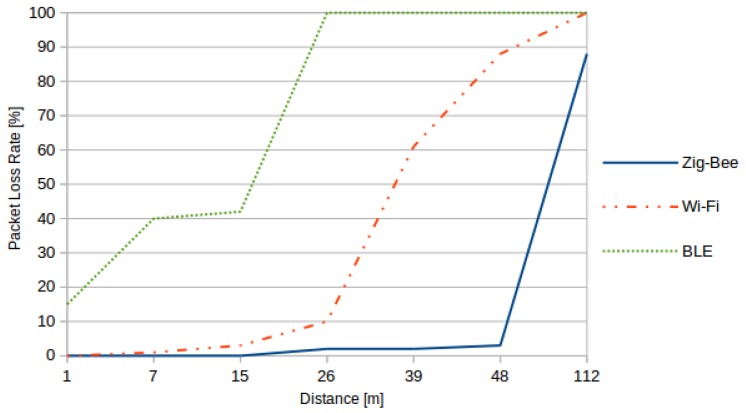
Impact of distance on the packet loss rate according to the considered protocols.

First, the reliability of protocols was tested by measuring the impact of distance on the packet loss rate. The packet loss rate is the ratio of packets lost to all packets sent. Results are shown in Figure 3.

**Figure 4:**
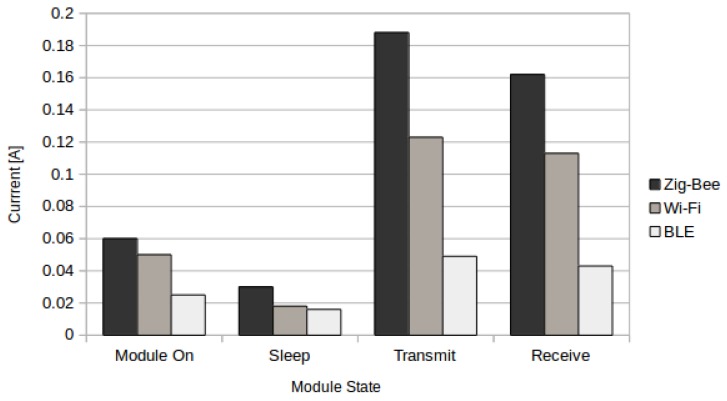
Current consumption of different protocols.

Battery consumption was also tested by measuring the current consumption of the protocols in different states. The results can be seen in Figure 4. ZigBee was the highest consuming protocol, capable of draining the battery of the system in 49 h.

The ZigBee module has the best measured range. With respect to Wi-Fi and BLE (which are common features of smart-phones), it should also be noted that ZigBee devices are less common among the population, thus providing a degree of protection against malicious data manipulation. The ZigBee module inside the Waspmote is a XBee-ZB-PRO S2, with the following characteristics:

- Supply voltage: 3.0–3.4 V
- Transmit power output: 50 mW (+17 dBm)
- RF data rate: 250,000 bps
- Data throughput: up to 35,000
- Serial interface data rate: 1200 bps–1 Mbps
- Operating frequency band: ISM 2.4 GHz.

Tests showed that with a continuous monitoring of sensors and the transmission of these data, the protocol with the least operational life was ZigBee, with a total of 60 h. However, this time span is enough to cover the expected duration of bicycle trips, In fact, supposing that the sensors could be mounted on the public bicycle sharing system and assuming that the bikes will be in their docking stations for extended periods of time, the battery consumption of the different protocols becomes irrelevant for the choice of the communication protocol. In conclusion, the ZigBee communication protocol was selected to be implemented in the proposed system.

## 5. Calibration

The collaboration with ARPA Piemonte allowed the tuning and calibration of the PM${}_{10}$ and O${}_{3}$ sensors. Thanks to this collaboration, ARPA granted access to their static stations and the data acquired by those stations in order to obtain reliable reference measurements. The objective of this operation was to verify the precision of the collected data by the mobile sensors and to calibrate the sensors based on ARPA reference data [32].

**Figure 5:**
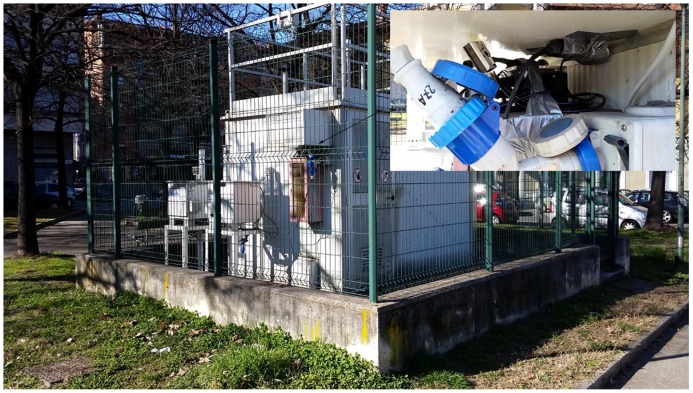
Test site at Rebaudengo Square in Torino. ARPA, Agenzia Regionale per la Protezione dell’Ambiente del Piemonte.

The first phase concerned the calibration of the PM${}_{10}$ sensor. ARPA Piemonte provided access to the data collection site at Rebaudengo Square, in Torino, as shown in Figure 5: the installed sensor can be seen at the top right. The device for the measurement of the PM${}_{10}$ was placed inside the structure. The PM${}_{10}$ data from ARPA Piemonte were provided daily. The data were collected via ZigBee protocols, downloaded and stored in the main servers of the Department of Control and Computer Engineering (at the Politecnico di Torino) for post-processing.

**Figure 6:**
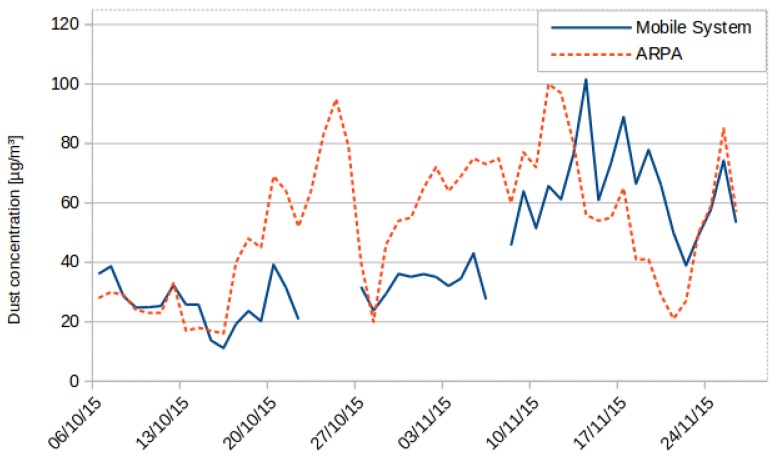
PM${}_{10}$ measurements at Rebaudengo Square. Lines correspond to the daily weighted mean.

Figure 6 shows a comparison between the average of the PM${}_{10}$ level measurements of all of the readings collected in a day by the proposed mobile node (in continuous-line) and the measurements of the PM${}_{10}$ levels measured by the sensor and from the ARPA sensors unit (in dashed-line) at Rebaudengo Square. The data obtained by the sensors were calibrated by using a linear interpolation method. This method implements a correction factor for the variance data and an offset for the mean of the readings.

$$C\left[PM_{10}\right]=\bar{ARPA}+\left(MV-\bar{MV}\right)\frac{\left(\sigma_{ARPA}\right)}{\left(\sigma_{MV}\right)}$$

$C[PM_{10}]$ corresponds to the corrected measurements after calibration; $\bar{ARPA}$ corresponds to the mean value of the data obtained by ARPA; while $\sigma_{ARPA}$ is the standard deviation of the same. *MV* is the voltage value measured by the mobile system. The formula for this particular sensor is:

$$C[PM_{10}]=1631.37\cdot MV+7.86$$

It can be noted that the mobile sensor is able to detect the increase and decrease in the values of the particulate matter presented. The measurements from the proposed system and the measurements from ARPA had a Pearson correlation factor of 0.61. Additionally, it has to be mentioned that the data had to be filtered due to the presence of outliers in the data for PM${}_{10}$; this is because of the nature of the scatter technique implemented in the sensor, where dust particles placed in front of the light emitting source of the sensor (an LED in this case) would block a larger portion of the light emitted, giving a higher reading than normal. A variable low-pass filter was applied during post-processing, and each data point was set to the average of the last three data points when it exceeded that average by more than three times.

**Figure 7:**
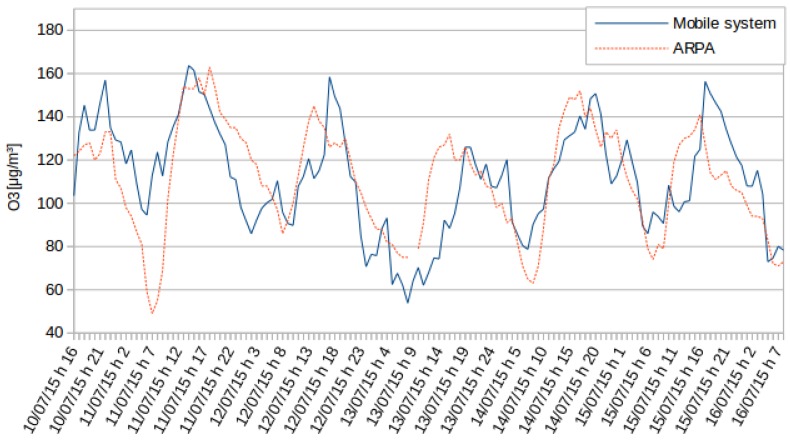
O${}_{3}$ measurement in the test site in Vinchio. The continuous line corresponds to the readings made by the mobile device, while the dashed line to the measurements gathered by ARPA.

The second phase involved the ozone sensor calibration. ARPA Piemonte provided access to the site data in Vinchio. Inside the structure, a device for the measurement of ozone was placed. The ozone data from ARPA Piemonte are provided hourly. Although a higher frequency would allow greater accuracy, the data acquired proved consistent with ARPA reference data. Data were acquired from sensors following the same protocol used for the station of Rebaudengo square. The ozone measurements of the mobile system are directly proportional to the internal resistance of the sensor. The values provided by the mobile device have been normalized with respect to those detected by ARPA Piemonte, adding a calibration factor in agreement with the average and the variance. The normalized data are shown in Figure 7. It should be noticed that the mobile sensor responds correctly to the daily rising edges and falls. Furthermore, the measured levels are quite similar to the ones from ARPA, with a Pearson correlation factor of 0.63. Consequently, the sensor measurements are not as accurate as the ones from ARPA, but they provide insight into the concentration of O${}_{3}$ in the area of interest.

A similar calibration approach was implemented in the case of the ozone sensors, with the following calibration formula:

$$C[O_{3}]=1.05*MV+110.82$$

Overall, the system presents a good accuracy with regards to the data acquisition, as confirmed by the high value of the Pearson correlation factor. Nevertheless, errors in readings are not negligible, and calibration factors are necessary.

## 6. Control Tests

The platform was tested in order to evaluate the characteristics of the different individual sensors and the system as a whole. In order to verify the validity of the measurements in motion, two tests were performed. An additional test was performed in order to check the performance of the sensors during prolonged periods of movement.

### 6.1. Tests of Multiple Sensors

The purpose of this activity is to check the repeatability of the proposed monitoring system. The test exploited multiple sensors of the same type, and the output is expressed in volts in order to compare raw data from the sensors.

**Figure 8:**
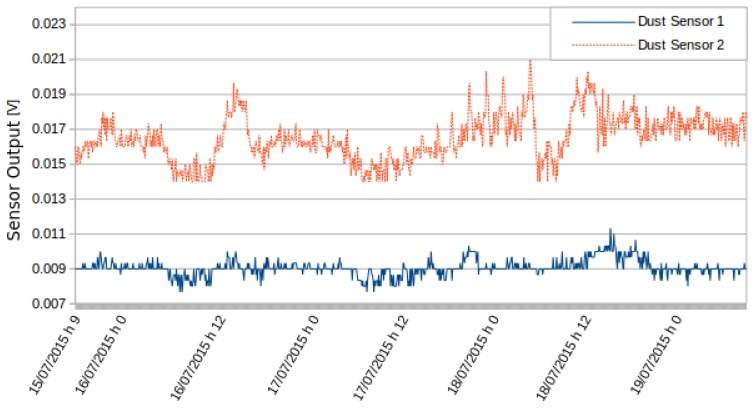
Measurements by two different PM${}_{10}$ sensors.

**Figure 9:**
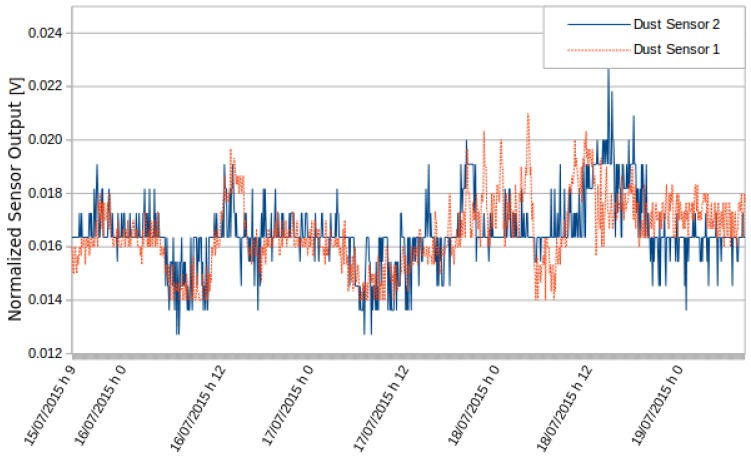
Normalized readings of the two different PM${}_{10}$ sensors.

In the first test, two PM${}_{10}$ sensors were used simultaneously in a static station, located at the Politecnico di Torino. Figure 8 shows the values detected by the two sensors. The values were consistent with the low variations of PM${}_{10}$. Figure 9 is obtained by normalizing over the variance the data at the average. Results show that the sensors detected the same variations and behaved consistently.

**Figure 10:**
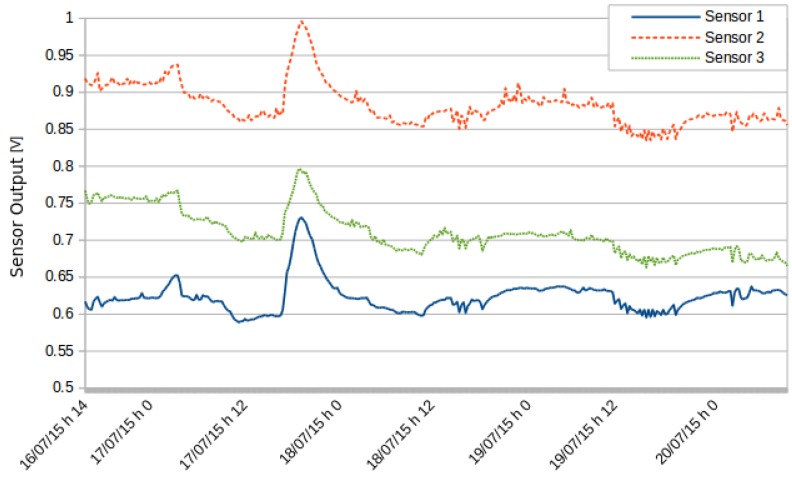
Measurements by three different O${}_{3}$ sensors.

**Figure 11:**
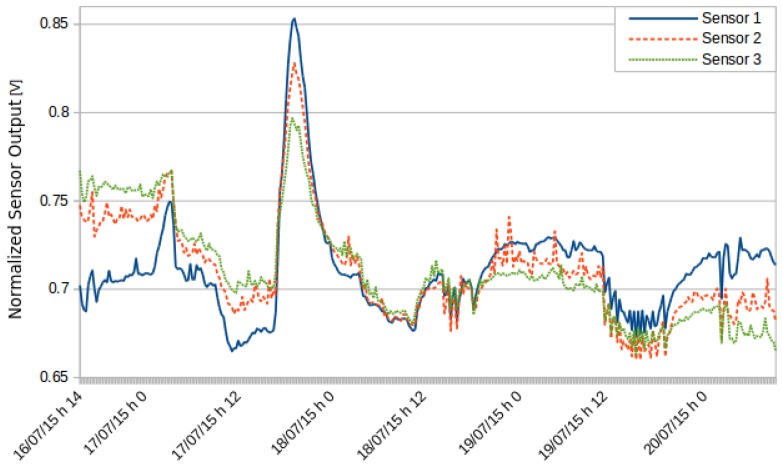
Normalized readings of the three different O${}_{3}$ sensors.

A second test involved three ozone sensors. Figure 10 shows the levels recorded by the three sensors. All sensors detected the same variations. Figure 11 shows the same data normalized with respect to the average and the variance. The ozone sensors have a small drift over time; therefore, calibration must be issued regularly whenever working with this type of sensor.

From the graphs and data presented so far (including Section 5), we can deduce that the sensors provide less accurate data than the systems available in ARPA, even after efficient calibration and normalization phases. However, sensors present some variance over time, thus the need for an automatic calibration program.

### 6.2. Mobility Tests

#### 6.2.1. First Mobility Test

The PM${}_{10}$ sensing device was transported by bicycle by alternating static and moving periods in order to verify if movement affects precision.

**Figure 12:**
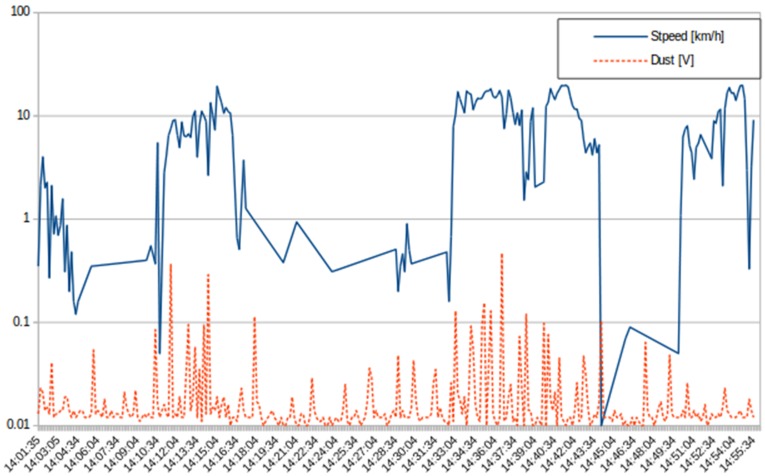
PM${}_{10}$ measured according to speed in the metropolitan area close to Politecnico di Torino.

This test was carried out by placing a PM${}_{10}$ sensor in a protective case against sun and wind, then mounting it on a bicycle. The measurements were taken in the metropolitan area adjacent to the Politecnico di Torino. Figure 12 shows the level of PM${}_{10}$ and the speed: the level of PM${}_{10}$ is represented with a continuous-line, while the dashed-line represents the speed. Given the values on the last part of the graph (after 14:50), it is possible to infer that an increase on the movement does not have a cumulative effect on the concentration of dust in the sensors.

#### 6.2.2. Second Mobility Test

**Figure 13:**
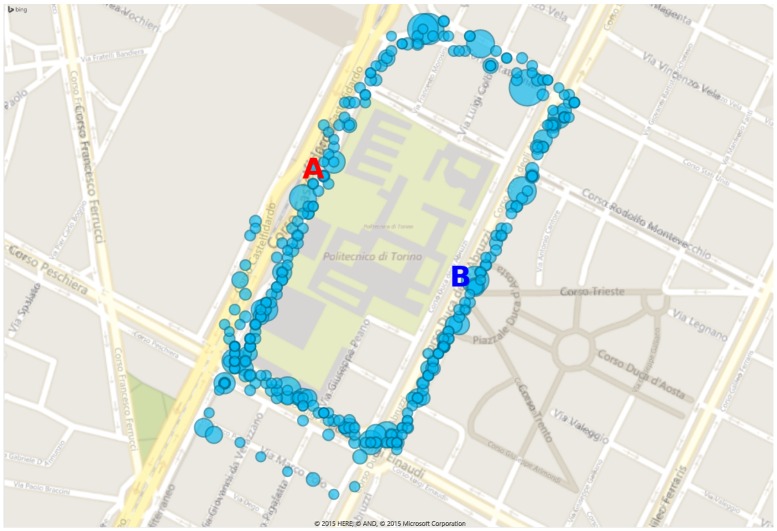
Path taken during measurements in the metropolitan area close to Politecnico di Torino.

In this test, static points of measurement were implemented, and a PM${}_{10}$ sensor was moved to check the impact of correlation between mobile and stationary measurements. The sensors used in this test are listed in Table 2. In detail, the test involved two fixed stations and one mobile; all sensors for a particular pollutant correspond to different sensors of the same model. The first fixed station was placed in Castelfidardo Avenue and contained an ozone sensor. The second location was set in Duca degli Abruzzi Avenue and contained an O${}_{3}$ and a PM${}_{10}$ sensor. Further PM${}_{10}$ and O${}_{3}$ sensors were mounted on a bicycle. Figure 13 shows the path covered during the test. The diameter of the circles indicating the position of the bicycle are directly proportional to the readings from the bicycle. This path was recreated using the GPS reading obtained with the internal GPS sensors of the node. The internal GPS sensor allows associating each measurement to the respective latitude, longitude and elevation of the location. There was no significant difference between the measurements made when moving or when the bicycle was stationary. Additionally, it is observed that the greater concentrations of PM${}_{10}$ are detected close to the entrances of the campus and close to crossroads, *i.e.*, where many cars stop with the engine running, as can be seen in Figure 13.

**Figure 14:**
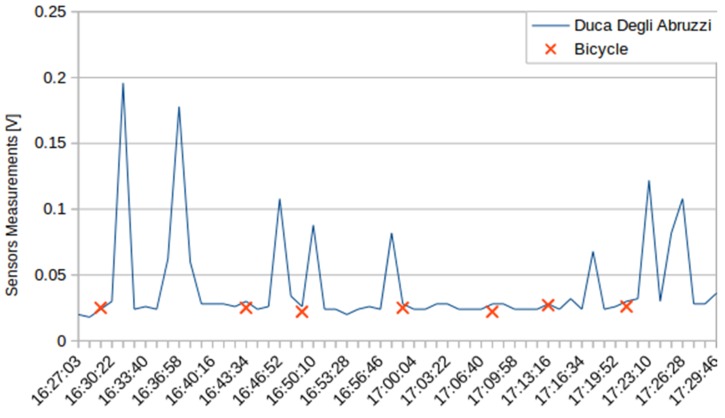
Comparison between fixed and mobile sensors.

The tests showed that measurements made on the move do not suffer significant alterations compared to those made at fixed locations. This can be seen in Figure 14, where the blue line corresponds to data collected by sensors in the fixed station at Duca Degli Abruzzi Avenue, and the red crosses correspond to the measurements of the sensors carried on the bike when passing close to the fixed station.

#### 6.2.3. Long-Run Mobility Test

**Figure 15:**
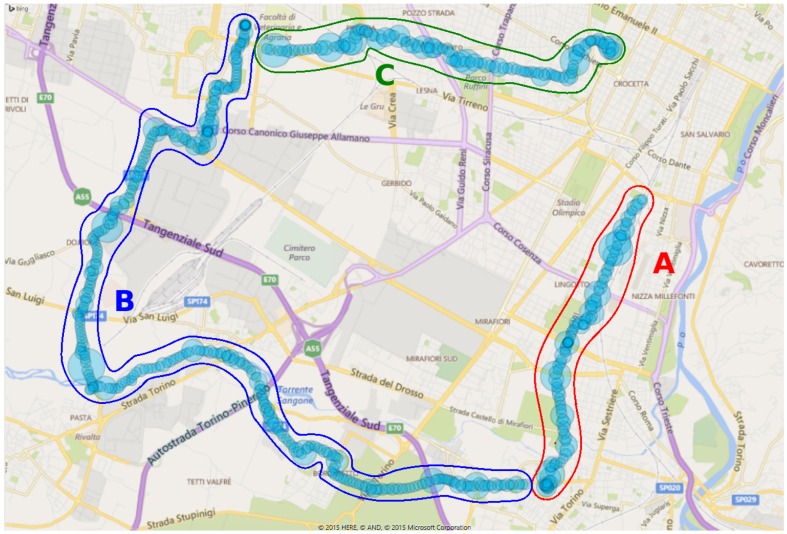
Path made across different municipalities in the metropolitan area of Torino.

A third test was carried out on long journeys in order to evaluate the mounting and robustness of the system, to evaluate shock impact, energy consumption, general reliability, temperature robustness, vibrations, humidity, *etc*. During this test, the PM${}_{10}$ sensor was mounted on a bicycle and traveled from the city of Torino to some neighboring municipalities. Figure 15 shows the path monitored during the test. Once again, the diameter of the circles on the map is proportional to the levels of PM${}_{10}$ readings made by the sensors. The experiment began at the Politecnico di Torino and involved an initial shift to Nichelino (segment marked with the letter A), then after a pause, the bicycle moved to Grugliasco (segment marked with the letter B), and then, the next morning, a new movement took place arriving at the Politecnico di Torino (segment marked with the letter C). The total distance covered was 30 km. Prolonged use of the device in motion did not reveal particular problems. During the test, the node was always powered by an internal battery.

**Figure 16:**
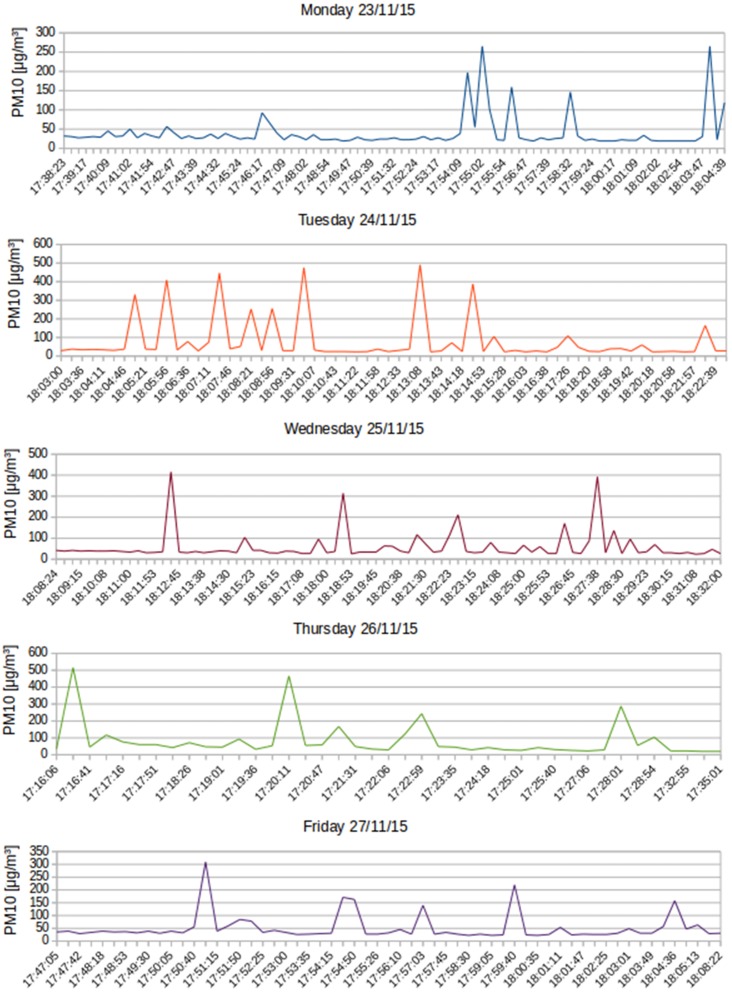
PM${}_{10}$ measurements from mobile sensors over five consecutive days.

For the last experiment, the same route was traveled over five consecutive days. In this experiment, a PM${}_{10}$ sensor was mounted on the bicycle. The route corresponds to the last part of the path shown in Figure 15 (Segment C), but this time, the starting point was the Politecnico di Torino (east point of the C section), across a busy urban area, followed by an interurban sector and ending in a suburban town: the length of the course is 7.1 km. In Figure 16, it can be observed that, every day, the curve representing the PM${}_{10}$ has a similar trend; with a decrease in PM${}_{10}$ concentration in the latter part of the measurements. The five tests were executed during the rush hour on a congested road. Data in Figure 16 were post-processed with the filter described in Section 5.

**Figure 17:**
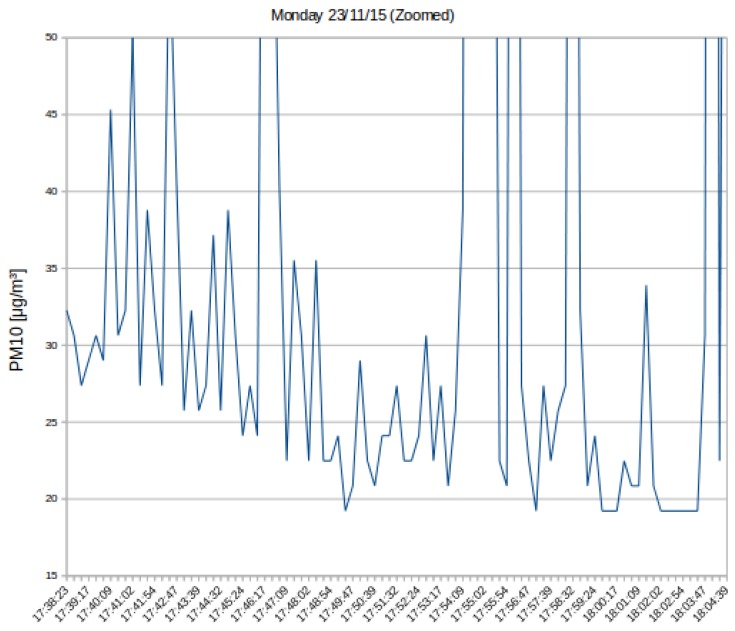
Zoomed PM${}_{10}$ measurements on the first day of the tests.

Figure 17 corresponds to a zoomed version of the first day of test from Figure 16. It can be seen the decreasing trend indicating that the further the distance from the city center, the lower the concentration of PM${}_{10}$.

**Figure 18:**
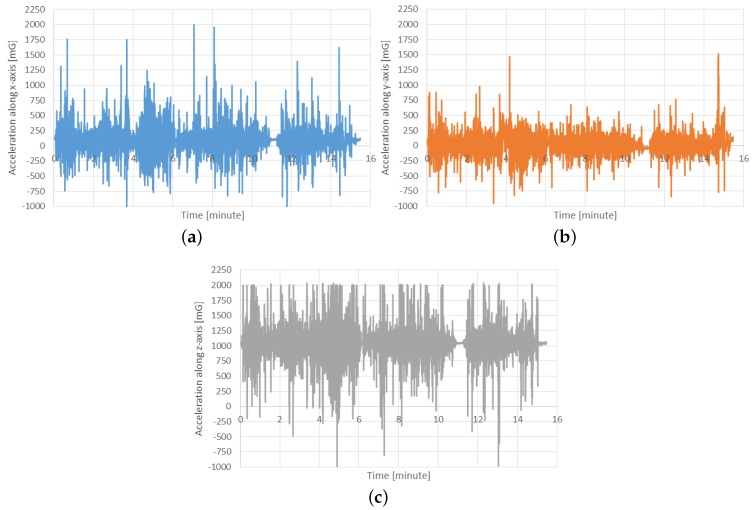
Acceleration values measured during a mobile test. (**a**) x-axis; (**b**) y-axis; (**c**) z-axis.

The analysis carried out has allowed a validation of the adopted sensors on the move, with a good consistency between static and on-the-move monitoring. The presence of erroneous data is not significantly different between the static and dynamic conditions. The robustness of the sensor is satisfactory, since no faults were detected. Acceleration values were recorded during movement in order to quantify the shocks to which the sensor was subjected. As Figure 18 plots, the acceleration force is generally limited, as it ranges between −1 g and +2 g. The lateral acceleration, due to the changes of directions, is the lowest one, while the frontal, due to the slowdown and increase of velocity, and the vertical acceleration, due to jerks, are the strongest ones.

## 7. Conclusions

A mobile and low-cost system to monitor air quality is proposed to complement the existing official one. The proposed system is capable of expanding the reach of the official system and of achieving a measurement resolution down to the street level. The measurements are less accurate, but have the potential to provide insight into one of the most critical areas in the whole European Union in terms of air pollution. During monitoring, the measures can be allocated to a particular location with the implementation of a georeference system.

Future works will include an extended analysis of the implementation of the system and the addition of new sensors capable of measuring the concentration of other noxious elements and/or gases in the atmosphere, e.g., NH${}_{3}$, LPGs, SO${}_{2}$.

## Figures and Tables

**Table 1:** Sensors used in the second mobility test.

Platform	Radio Link	Programming Language	Power Supply	Cost
SunSpot	802.15.4	Java	Batteries + USB	˜€180
TelosB	802.15.4	NesC	Batteries + USB	˜€150
CM4000	802.15.4	NesC	Batteries + USB	˜€80
Waspmote Plug & Sense	802.15.4	C (Arduino standard)	Batteries + USB + Solar Panels	˜€500
	ZigBee			
	Wi-Fi (802.11b/g)			
	3G/GPRS			
	BLE			

**Table 2:** Sensors used in the second mobility test.

Station	Location	Sensors	Tag in Figure 13
Location A	Av. Castelfidardo	O${}_{3}$	A (Red)
Location B	Av. Duca degli Abruzzi	O${}_{3}$ and PM${}_{10}$	B (Blue)
Bike	Mobile	O${}_{3}$ and PM${}_{10}$	Teal circles
